# Spatial Deconstruction of the Plasma Membrane

**DOI:** 10.1021/jacs.6c10688

**Published:** 2026-08-04

**Authors:** Venkata Shiva Mandala, Chen Zhao, Qiuying Chen, Steven Gross, Jacob Geri, Roderick MacKinnon

**Affiliations:** † Laboratory of Molecular Neurobiology and Biophysics, Howard Hughes Medical Institute, 5929 The Rockefeller University, New York, New York 10065, United States; ‡ Department of Pharmacology, 12295 Weill Cornell Medicine, New York, New York 10065, United States

## Abstract

The plasma membrane of cells is known to be heterogeneous with regard to the spatial distribution of proteins and lipids, but the nature and origins of this heterogeneity are unclear. In this study, we perform fluorescence microscopy on plasma membrane sheets and find that most proteins occur in protein-rich domains separated by intervening protein-poor regions. We show that the protein-rich domains and protein-poor regions are at least partially preserved in plasma membrane-derived vesicles that lack cytoskeletal elements, permitting their separation and isolation by density centrifugation. Compositional analysis by nuclear magnetic resonance and mass spectrometry shows that different proteins and lipids exhibit distinct tendencies to partition into the protein-rich domains. This differential partitioning is correlated with the function of the different proteins, suggesting segregation based on cellular processes. Likewise, ordered lipids, including cholesterol and sphingomyelin, differentially segregate, being more abundant in the protein-rich domains. We propose that the collective assembly of certain proteins and lipids creates the heterogeneous distribution of membrane components and the emergence of protein-rich domains. These domains could create distinct environments for the function and segregation of various membrane processes.

## Graphical abstract

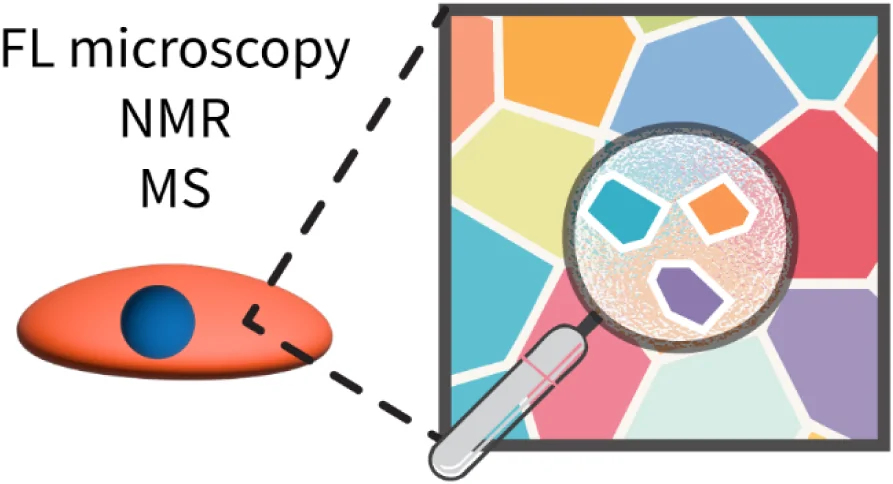

## Introduction

The plasma membrane of a living cell serves not only to insulate the cell from its environment but also as a staging for signaling processes to interact with the outside world.^1^ The organization of this membrane thus underlies many important biological processes, including signal transduction, cell–cell interactions, and nutrient transport. At the simplest level, the plasma membrane is a composite of proteins and lipids that assemble into an organized structure that supports the array of biological functions carried out in the membrane.^2^

It is well-known from microscopy and molecular tracking studies that the distribution of individual proteins and lipids in the plasma membrane is heterogeneous.^3^ Various models have been proposed to explain this heterogeneity – protein–protein interactions,^4,5^ partitioning into membrane domains with specific properties,^6,7^ and segregation by cytoskeletal elements.^8,9^ Yet there is no consensus on the general principles of plasma membrane assembly and their underlying mechanisms.^2^

We became interested in this topic while studying signaling pathways in cardiomyocytes using immunolabeling. We observed that many membrane proteins – including G-protein coupled receptors, ion channels, and an enzyme – form self-specific clusters, termed higher order transient structures (HOTS), indicative of weak protein–protein interactions.^10^ These HOTS protein clusters did not appear in all regions of our micrographs but were rather concentrated in regions that appear electron dense in our uranyl-staining experiments. These regions, which we term “protein-rich domains” (and some others have called protein islands^11–17^ ) seemingly harbored most of the protein clusters that we studied.

At the same time, using cryo-electron microscopy we were also studying the structures of proteins in vesicles derived directly from the plasma membranes of cells.^18^ We noticed that these vesicles were highly heterogeneous in their appearance: some contained a high density of proteins, whereas others appeared relatively empty. We wondered whether the protein-rich vesicles were derived from the protein-rich domains observed in our immunolabeling experiments, and the protein-poor vesicles from the intervening protein-poor regions of the plasma membrane. If this were the case, the cell-derived vesicles could prove useful to isolate and study spatial domains present in the plasma membrane.

Here, the first set of experiments examines the existence of protein-rich domains in cell membranes. We used unroofed cell membranes and mildly fixed whole cells, which are subject to artifacts. We note, however, that others have observed patterns in living cells and in various other plasma membrane preparations that are consistent with those we observe.^10–16^ The second set of experiments establishes the persistence of relatively protein-rich and protein-poor domains in giant plasma membrane vesicles derived from the cell membrane. The third set of experiments analyzes the protein and lipid compositions of fractionated protein-rich and protein-poor vesicles derived from the larger vesicles, and thus from the plasma membrane. Our analysis leads us to conclude that most but not all membrane proteins are concentrated in protein-rich domains in the plasma membrane. A smaller subset of proteins with distinct functions are not concentrated but rather are more evenly distributed over the membrane. Further, lipids are also unevenly distributed, with sphingomyelin and cholesterol existing at higher concentrations in protein-rich domains.

These experiments demonstrate the existence of distinct domains in plasma membranes, how to isolate them, and how to characterize their composition. Our initial analysis shows that functionally connected proteins reside within domains, implying that they subserve membrane function.

## Results

### Protein-Rich Domains in Unroofed Membranes and in Intact Cells

To study the distributions of all membrane-associated proteins in the plasma membrane, we labeled proteins specifically but indiscriminately using a cysteine-reactive fluorophore and labeled lipids using a membrane-partitioning dye (Figure 1A). This targeted, indiscriminate labeling was carried out either on membrane sheets obtained by unroofing the cells or on intact, fixed cells (Figure 1). We used HEK293 cells grown on glass coverslips coated either with poly-d-lysine or both poly-d-lysine and laminin, as these same cells were used later in suspension culture to generate the cell-derived vesicles. Similar domains have been reported previously in cardiomyocytes, T cells, B cells, mast cells, PC12 cells, and in COS-7 cells grown on different substrates.^10–16^

**Figure 1:**
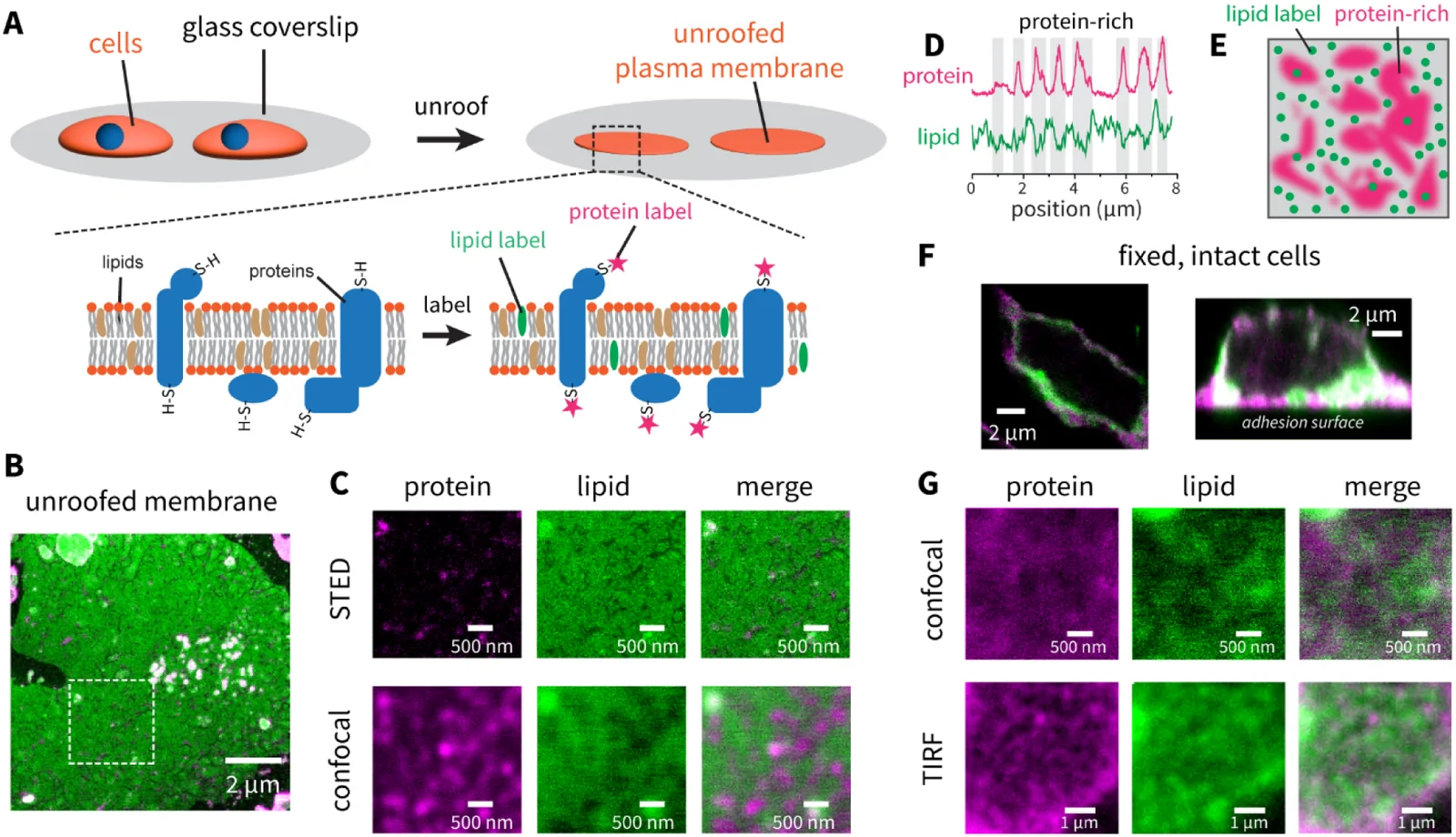
Labeling of proteins and lipids in plasma membranes reveals protein-rich domains. (**A**) Schematic of unroofing and labeling procedure. Cells are grown on a coated glass coverslip and then unroofed leaving behind membrane sheets. Proteins are nonspecifically labeled at cysteine residues using a maleimide-conjugated fluorophore (STAR 580-maleimide) and lipids are labeled by the addition of a membrane-partitioning dye (STAR RED-PEG2000-DPPE). (**B**) Representative STED images of unroofed membranes labeled for proteins (magenta; STAR 580-maleimide) and lipids (green; STAR RED-PEG2000-DPPE), showing relatively homogeneous lipid distribution but more punctate protein distribution. (**C**) Zoomed-in view of the square region from panel B, showing images acquired using STED (top) or confocal (bottom). (**D**) Representative intensity profiles for proteins (magenta) and lipids (green) along a line, showing anticorrelation of the signals. (**E**) Schematic showing the interpretation of the nonhomogeneous protein signal and the anticorrelation with the lipid signal, namely that there exist protein-rich regions at the plasma membrane that tend to exclude the lipid label. (**F**) Representative confocal images of fixed, intact cells labeled for proteins (magenta; STAR 580-maleimide) and lipids (green; STAR RED-PEG2000-DPPE), showing that the labeling is primarily at the plasma membrane. (**G**) Representative confocal (top) and TIRF (bottom) images of proteins and lipids at the basolateral membrane. The protein signal is unevenly distributed and coincides with regions of reduced lipid signal, as in the unroofed membranes. Proteins are labeled with STAR 580-maleimide and lipids are labeled with STAR RED-PEG2000-DPPE.

We first looked at protein and lipid distributions in plasma membrane sheets obtained by unroofing adherent cells (Figure 1A). We used shear flow for unroofing, but similar results were obtained by sonication following established protocols^19^ (SI Appendix, Figure S1). Figure 1B shows a representative stimulated emission depletion (STED) microscopy^20^ image of an unroofed cell membrane labeled for proteins (magenta) using STAR 580-maleimide and for lipids (green) using STAR RED-PEG2000-DPPE.^21^ While the lipid signal appears relatively homogeneous (with some variations that will be discussed), the protein signal is concentrated at certain spots rather than being a diffuse sheet. When looking at the protein and lipid signals more closely (Figure 1C), most of the bright protein spots tended to be in regions where the lipid signal was weaker. This anticorrelation between the two channels (Figure 1D) is accentuated in the STED data but is also visible in the confocal images (Figure 1C). Additional images from different biological replicates and labeling probes are given in SI Appendix, Figure S2.

What causes this anticorrelation between the protein and lipid signals? If we observed regions that are bright in both signals (which we do in some areas, see Figure 1B), the simplest explanation would be some sort of local membrane topological variation^22^ or vesicles that remain attached to the unroofed membranes, that would result in greater membrane signal and greater protein signal. But instead, we see “shadows” in the lipid signal that are *sometimes* bright in the protein channel. To exclude artifacts related to specific labels, we tested different fluorophores for the protein and lipid labels, lipid reagents with different chemical structures, and omitted the protein label entirely (SI Appendix, Figure S3), with similar results. We also ensured that the lighter and darker regions in the lipid signal are not a result of local membrane topology using *xyz* sectioning (SI Appendix, Figure S4).^23^

We interpret the anticorrelation of the protein and lipid signals (Figure 1D) to be due to “protein-rich domains” where lipids are partially excluded (Figure 1E), perhaps simply by volume occupancy (or by some other means such as lipid composition). This explanation accounts for why not all of the shadows in the lipid signal are bright in the protein channel, because some of these domains presumably have proteins that are not labeled with the cysteine-reactive dye. We point out that we are not the first to describe domains that fit our description here; others have noted similar domains in studies using electron microscopy, atomic force microscopy, and fluorescence microscopy,^11–16^ but have not discussed the anticorrelation between lipid and protein labels.

What is the nature of these protein-rich domains? Some domains appear “stringy” while others appear roughly circular and both are presumably diffraction limited. The stringy appearance of some domains naturally raises the question of cytoskeletal elements. To test this, we labeled the unroofed membranes with actin stains (SI Appendix, Figure S5) and found that these domains are only very sparsely labeled with actin markers, while actin labeling in intact cells was as expected. Furthermore, the fact that the lipid signal is depleted in these regions suggests that the proteins are present within the membrane, as proteins in the cytosol would not be expected to cause local depletion of the lipid stain. We thus conclude that both the stringy and the circular domains likely correspond to integral or peripheral membrane proteins, and perhaps the stringy domains represent proteins that tether the cytoskeleton to the cell membrane.

Do these protein-rich domains exist in intact cells, or are they an artifact of unroofing? To test this, we needed reagents that primarily label proteins and lipids at the plasma membrane but not inside the cell. We found that labeling for 5–10 min in serum-free media using STAR 580-maleimide (to label proteins) and STAR RED-PEG2000-DPPE (to label lipids),^21^ followed by chemical fixation using PFA and then mounting of the coverslips resulted in staining primarily at the plasma membrane of these cells (Figure 1F). We then imaged the plasma membrane at the adhesion surface either using a confocal microscope or using a total internal reflection fluorescence (TIRF) microscope (Figure 1G) to examine protein and lipid distributions. The protein signal and lipid signal were anticorrelated in the cells, consistent with our findings in the unroofed membranes and with other studies that have shown their existence in live cell experiments.^12^ Thus, the plasma membrane of cells appears to be organized into protein-rich domains and protein-poor domains.

### Protein-Rich Domains Persist in Giant Plasma Membrane-Derived Vesicles

Are these protein-rich domains only present in intact cells, or can they be isolated? We wondered whether they persist in cytoskeleton-free giant plasma membrane-derived vesicles (GPMVs), which are derived from cells by treatment with N-ethylmaleimide (NEM) and calcium (Figure 2A, B).^24,25^ GPMVs were isolated from cells (Figure 2A, B) and burst onto the same glass coverslips used for the cellular imaging experiments. The membrane sheets were labeled using a membrane-partitioning dye (CellMask Orange). Protein labeling was attempted but was unsuccessful, presumably due to NEM-capping of free cysteines. However, we reasoned that we could simply look at the variation in the lipid signal to infer the presence of protein-rich domains, (Figure 2C), as we just concluded that in cells the lipid signal is anticorrelated with the protein signal (Figure 1D, E). These experiments were carried out at ∼20 °C, well above the temperature at which macroscopic phase separation occurs in these types of GPMVs.^25^ We note that since we are unable to directly observe the proteins in this experiment, inhomogeneous dye partitioning, local curvature differences, or bursting/surface-adhesion artifacts could contribute to lipid signal variation in GPMVs.

**Figure 2:**
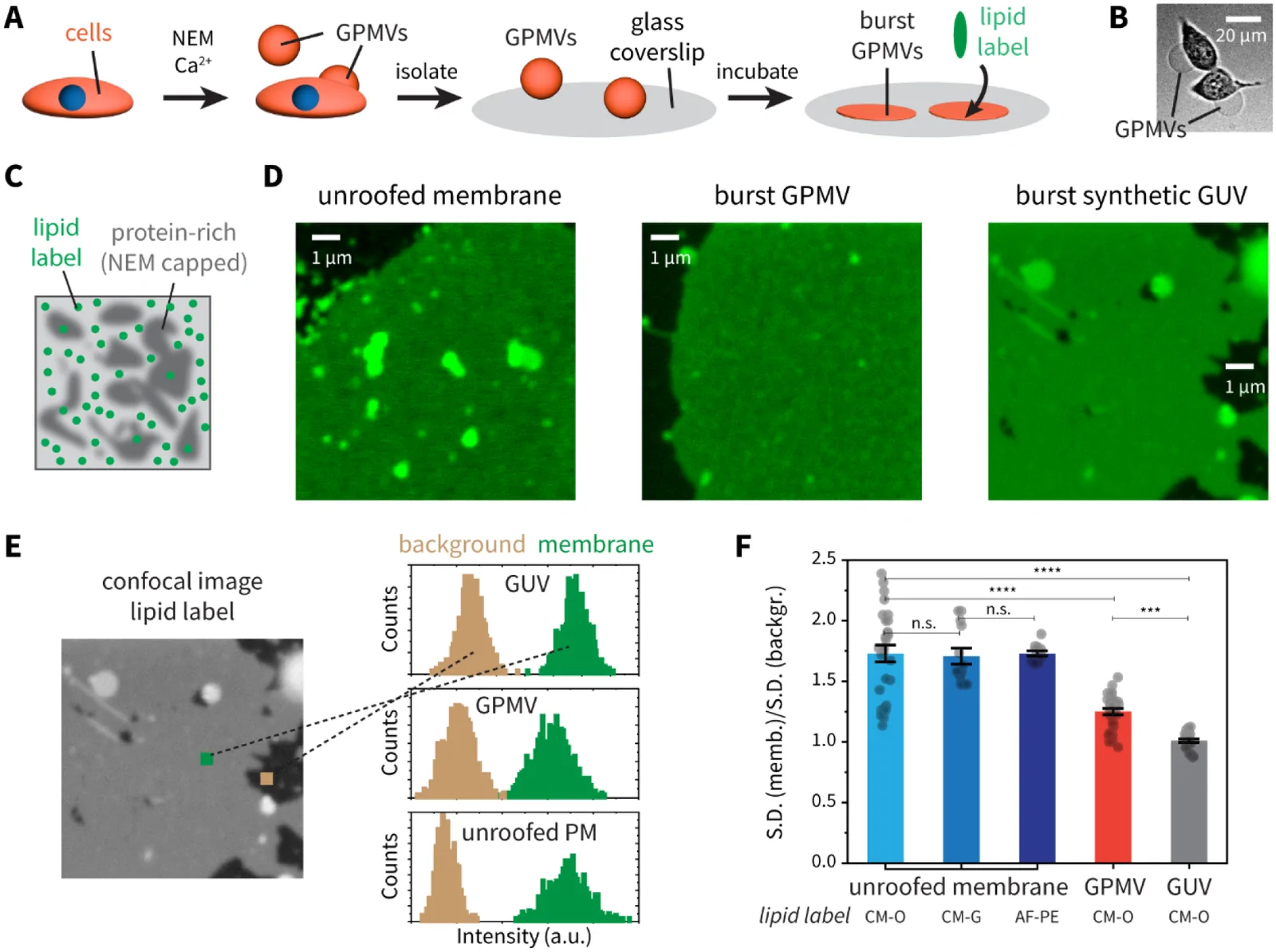
Protein-rich domains are also present in giant plasma membrane-derived vesicles. (**A**) Schematic of giant plasma membrane-derived vesicle (GPMV) generation from cells and their subsequent bursting for imaging. (**B**) Brightfield image of GPMVs budding off from cells. (**C**) Schematic showing the burst GPMV labeling method. As free cysteines are capped during NEM treatment, only the lipid label can be used and thus the protein-rich domains cannot be directly imaged using fluorescence microscopy. However, the presence of these domains can be inferred from the inhomogeneity of the lipid signal, which does not label the protein-rich domains as well as it does the protein-poor domains. (**D**) Representative confocal images of unroofed membranes from cells (left), burst cell-derived GPMVs (middle), and burst synthetic GUVs (right), all labeled using the same lipid partitioning label. (**E**) Principle of signal heterogeneity analysis in the membrane sheets: the width of the distribution of intensities in the background is compared to the corresponding distribution inside the membrane sheet. If the distributions are of similar width, then the signal heterogeneity can be explained simply by noise. (**F**) Plot of the ratio of the standard deviations of the distribution inside the membrane over that in the background (outside the membrane), a measure of the signal heterogeneity inside the membrane. If the ratio equals 1 (as is the case for GUVs), then the signal from the lipid label is taken to be homogeneous within the membrane. The data for unroofed cell membranes using three different lipid labels (CellMask Orange, CellMask Green, and AF594-PE) are shown. CellMask Orange was used for the experiments with GPMVs and GUVs. The box represents the mean for each sample and the error bar is the standard error of the mean. The statistical test used was Welch’s *t* test.

Figure 2D shows representative results of lipid staining in unroofed plasma membranes, in burst GPMVs, and in lipid-only GUV controls. Variation in lipid signal intensity can clearly be seen in the unroofed membranes and in the burst GPMVs but is not visible in the GUV samples. To ensure that this phenomenon is not simply an optical illusion, we measured the distribution of intensities in small regions (chosen to not have any gross bright or dark spots) taken from either the membrane region or from the background in these images (Figure 2E). The idea is that there is some natural variation in intensities due to noise. In the case of GUVs, the width of the distribution of intensities (a measure of the range of intensities in that region) is similar in the membrane sheet and in the background (Figure 2F). Meanwhile in unroofed membranes and in GPMVs the signal from the membrane is noticeably broader than the background (Figure 2F), but GPMVs show reduced variation compared to unroofed membranes. With the caveat that in this experiment we do not directly image proteins, we conclude that protein-rich domains likely persist to some extent in GPMVs. GPMVs are known to be free of cytoskeletal elements.^24,26^ Thus, protein-rich domains likely arise spontaneously from the interactions within the mixture of proteins and lipids in the cell membrane.

### Purification of Protein-Rich and Protein-Poor Vesicles

The data presented above, aligning with other studies,^11,12^ suggest that the cell membrane is organized into protein-rich domains. The data presented in this paper also indicate that protein-rich domains persist to some degree when isolating giant vesicles from cells. This finding suggests a way to isolate and analyze protein-rich and protein-poor regions from the cell membrane. After washing cells to remove secreted vesicles in the media, we generated GPMVs as described in methods and then gently sonicated them to produce smaller (submicron sized) vesicles we call small plasma membrane vesicles (SPMVs) (Figure 3A). The size of SPMVs (∼50–100 nm in diameter) is around the size of protein-rich domains observed in cell membranes (∼100 nm). Cryo-electron microscope (cryo-EM) images show that some of the vesicles are filled with proteins and others are relatively empty (Figure 3B). If proteins were randomly distributed in the membrane with, for instance, a Poisson distributed protein density, then the vesicles derived from the membrane should not show many nearly empty vesicles coexisting with many protein-packed vesicles. Why? Because for a random distribution of proteins, the presence of many protein-packed vesicles would predict very rare empty vesicles. By contrast, a cell membrane with protein-rich and protein-poor regions would be expected to generate vesicles with a multimodal distribution of protein density, as we see in cryo-EM micrographs.

**Figure 3:**
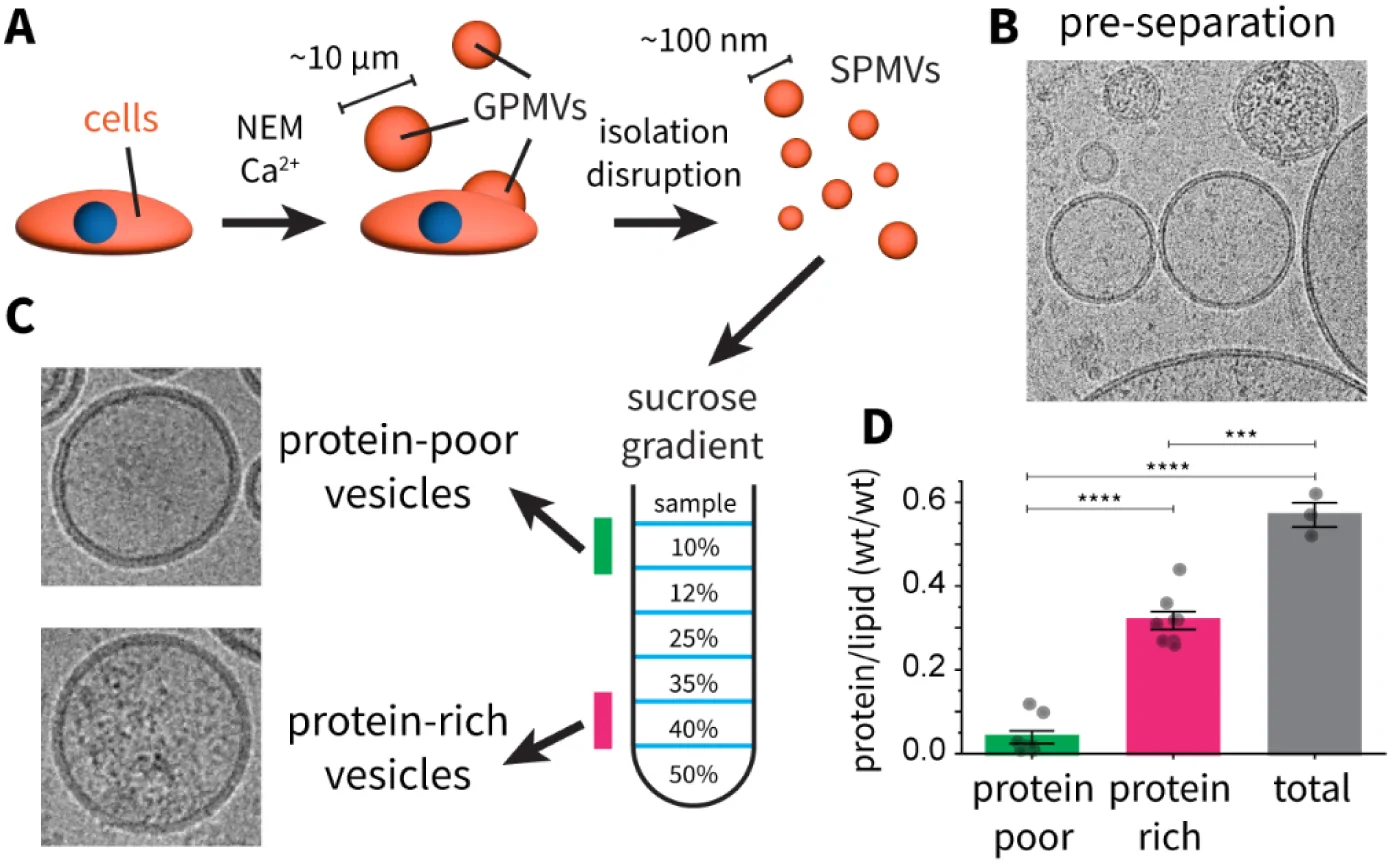
Isolation of protein-rich and protein-poor vesicles. (**A**) Schematic of small plasma membrane-derived vesicle (GPMV) generation from cells and their subsequent separation using a sucrose density gradient. (**B**) Cryo-EM image of cell-derived vesicles before separation, showing the presence of both vesicles with lots of protein and vesicles with little protein in them. (**C**) Representative cryo-EM images of protein-poor (top) and protein-rich (bottom) vesicles after separation using a sucrose density gradient. (**D**) Protein-to-lipid ratios (w/w), of the protein-poor, protein-rich and unseparated samples. The unseparated sample has a greater protein-to-lipid ratio because the bottom, most dense fractions from the sucrose gradient are not used. Protein concentration was calculated using a BCA assay and lipid concentration using ^1^H NMR. The box represents the mean for each sample and the error bar is the standard error of the mean. The statistical test used was Welch’s *t* test.

The critical reader would be correct to wonder whether the protein-rich vesicles (Figure 3B, C) are rich in membrane proteins or cytoplasmic proteins. We show in SI Appendix, Figure S6A, that membrane proteins can have the appearance of being inside the vesicles rather than on the surface. But inspection of the membrane on the edge of vesicles shows that protein-rich vesicles contain many membrane proteins. Furthermore, we analyze (below) the protein composition of vesicles by mass spectrometry and find they contain membrane proteins and soluble proteins that are apparently associated with the membrane proteins. Consistent with this association, the abundance of membrane proteins versus total proteins is the same in the protein-rich and protein-poor vesicles (SI Appendix, Table S1). The important point is, the protein-rich vesicles are dense in membrane proteins.

To fractionate SPMVs into protein-rich and protein-poor vesicles we used sucrose density gradient ultracentrifugation (Figure 3C).^27^ There is, of course, a continuum of vesicle densities, but we chose fractions near the two extremes as representative examples (see SI Appendix, Figure S6B for representative example of a sucrose gradient). Cryo-EM imaging of the purified vesicles confirmed that the different fractions from the sucrose gradient were indeed protein-rich or protein-poor (SI Appendix, Figure S6C). To quantify the amount of proteins and lipids, we used a bicinchoninic acid (BCA) assay^28^ to measure total protein concentration in the different samples and ^1^H nuclear magnetic resonance (NMR) spectroscopy^29^ to quantify the total lipid concentration. The calculated protein-to-lipid ratios for the unpurified sample and the isolated protein-rich vesicles and protein-poor vesicles are shown in Figure 3D. The protein-rich vesicles have about 10-fold more protein per lipid surface area compared to the protein-poor vesicles, and the ratio of protein and lipid in the protein-rich and unpurified samples is similar to published values on the composition of the plasma membrane,^30–32^ i.e., that proteins and lipids are about equal in abundance. The total amount of lipids is similar in the two fractions; thus, the protein-rich and protein-poor domains occupy roughly similar areas on the plasma membrane.

The above observations are consistent with a hypothesis that protein-rich and protein-poor vesicles are derived from protein-rich and protein-poor domains on the cell membrane (Figure 4A). We next analyze the composition of these different vesicles.

**Figure 4:**
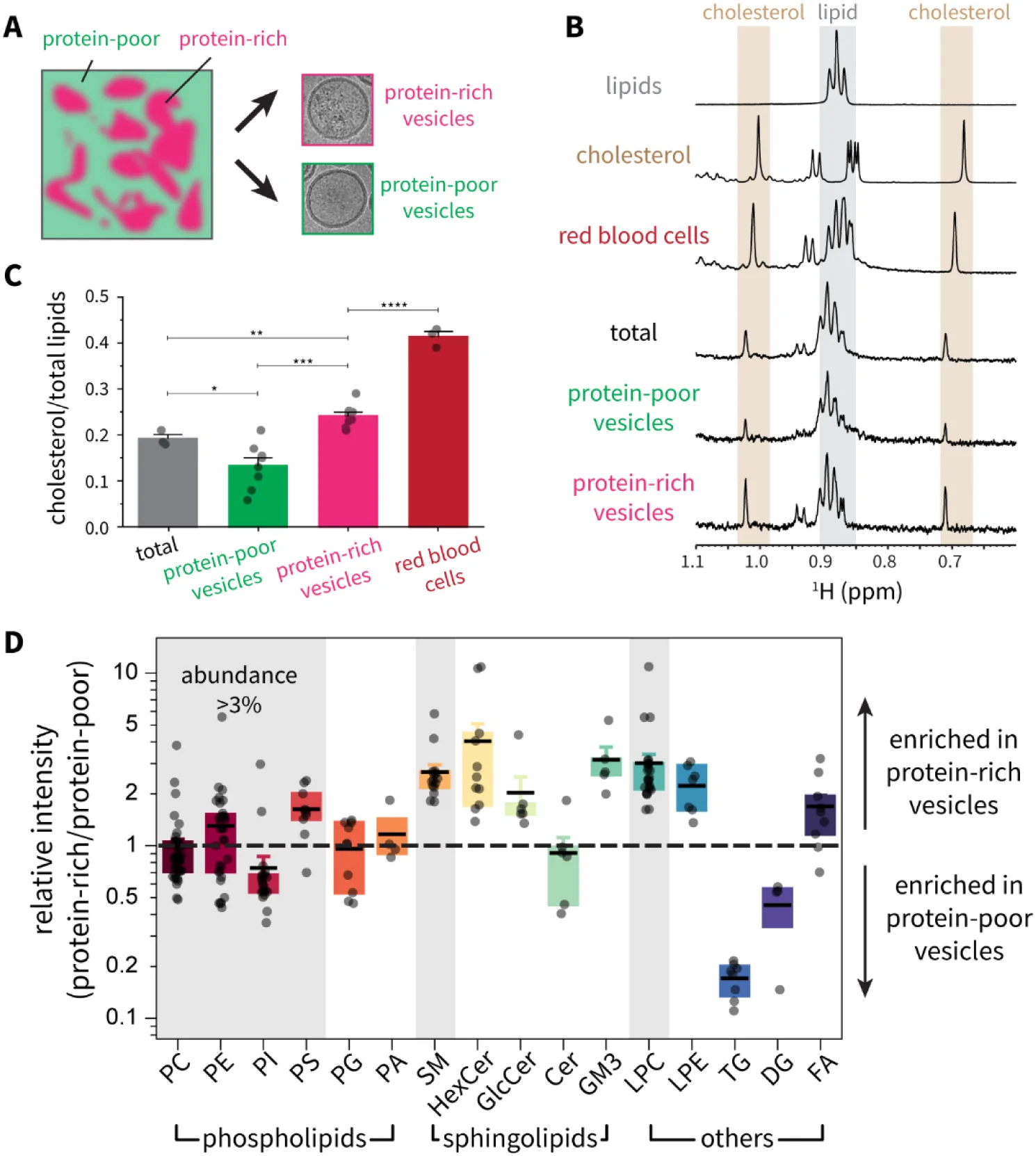
Lipid and cholesterol content of protein-rich and protein-poor domains. (**A**) Schematic of protein-rich domains and protein-poor domains at the plasma membrane, which are isolated as protein-rich and protein-poor vesicles. (**B**) Determination of lipid and cholesterol content using ^1^H NMR. The methyl region of the spectrum is shown with two resolved methyl signals from cholesterol and the methyl triplet from the terminal carbon in lipids. The top two spectra are from pure phospholipids and from cholesterol, respectively. (**C**) Cholesterol-to-lipid ratios (mol/mol) of the protein-poor, protein-rich and unseparated samples, and that of red blood cells determined using the same method as a control. The box represents the mean for each sample and the error bar is the standard error of the mean. The statistical test used was Welch’s *t* test. (**D**) Relative intensities of different lipid species in the protein-rich vesicles divided by their intensities in the protein-poor vesicles, from lipid mass spectrometry. Lipid species are organized by headgroup, and each dot represents a unique lipid (with the indicated headgroup and a unique combination of fatty acid tails). Headgroups with the highest abundance according to mass spec intensity are marked with the gray shaded boxes. The box represents the middle 50% of the data (25–75% percentile range) and the error bar is the standard error of the mean.

### Cholesterol and Ordered Lipids Are Enriched in Protein-Rich Domains

Lipid heterogeneity is thought to be important for plasma membrane organization *in vivo*. We thus measured lipid compositions in the two sets of vesicles. We measured cholesterol and total lipid content using ^1^H NMR as it is highly quantitative. The methyl region of the spectrum (Figure 4B) shows the triplet from the terminal methyl of fatty acid chains in lipids, as well as two resolved methyl signals from cholesterol. We used the ratio of these lipid and cholesterol signals to calculate the fraction of cholesterol (cholesterol/total lipids) in the protein-rich vesicles, protein-poor vesicles, and in unenriched SPMVs as approximately 25%, 13%, and 20%, respectively (Figure 4C). The cholesterol content in protein-rich vesicles is thus about 2-fold higher than that in protein-poor vesicles. We performed similar recordings on red blood cells as a test of the accuracy of our method. We found a cholesterol fraction of about 40% (Figure 4B, C), in line with established reports on red blood cells.^33^

To quantify other lipid species we used mass spectrometry. The relative enrichment of discrete lipid species (distinguished by their acyl chains), grouped by headgroup, in the protein-rich and protein-poor vesicles is shown in Figure 4D. Lipids that are more ordered, such as sphingomyelin (SM), hexosylceramides (HexCer), and gangliosides (GM3), as well as phosphatidylserine (PS) are enriched in the protein-rich vesicles, whereas phosphatidylcholine (PC) and phosphatidylinositols (PI) are more abundant in the protein-poor vesicles, and phosphatidylethanolamine (PE) is equally distributed. We also analyzed enrichment across chain length and unsaturation (SI Appendix, ) and found no clear trend among the different head groups. Cholesterol amounts were not measurable in these lipidomics experiments, but the NMR data addressed cholesterol. The enrichment of cholesterol in these domains raises the question whether depleting cholesterol disrupts these domains. Others have shown that cyclodextrin-mediated depletion of cholesterol not only disrupts these domains, but that the addition of exogenous cholesterol can promote the formation of these protein-rich domains.^12^ In summary, the protein-rich vesicles appear enriched in ordered lipids as well as cholesterol. A discussion of this finding in the context of the lipid raft hypothesis will follow later.

### Different Proteins Exhibit Differential Partitioning into Protein-Rich Domains

If protein-rich vesicles originate from protein-rich domains, then we might expect there to be some differential segregation of certain proteins toward protein-rich domains and other proteins not. For example, some proteins might function in the crowded environment of a protein-rich domain, whereas others might function in a more dilute (protein-poor) environment. To address whether differential segregation exists, we performed bottom-up proteomics measurements on protein-rich and protein-poor vesicles normalized by the measured lipid and protein concentrations to account for the different protein density (per surface area) in the two fractions. Raw intensities for proteins were corrected within each sample using variance stabilizing normalization (VSN).^34^ The normalized intensities were then corrected by the known protein and lipid concentrations (measured using the BCA assay and ^1^H NMR respectively; Figure 3D) for each sample to adjust for the area of the cell membrane. The partition ratio (PR) for each protein was calculated as the ratio of the intensity of that protein in the protein-rich vesicles divided by the intensity of the same protein in the protein-poor vesicles (Figure 5A), averaged over all pairwise sets of samples. For each protein with a PR, we mined the UniProt database^35^ to obtain annotations for protein subcellular localization. Using this approach, we calculated partition ratios (PRs) for 4,258 proteins, of which about half are membrane proteins of different classes – multipass, single-pass, lipid-anchored and peripheral (Figure 5B). The cytoplasmic proteins that were identified span broadly the cytoplasmic proteome.

**Figure 5:**
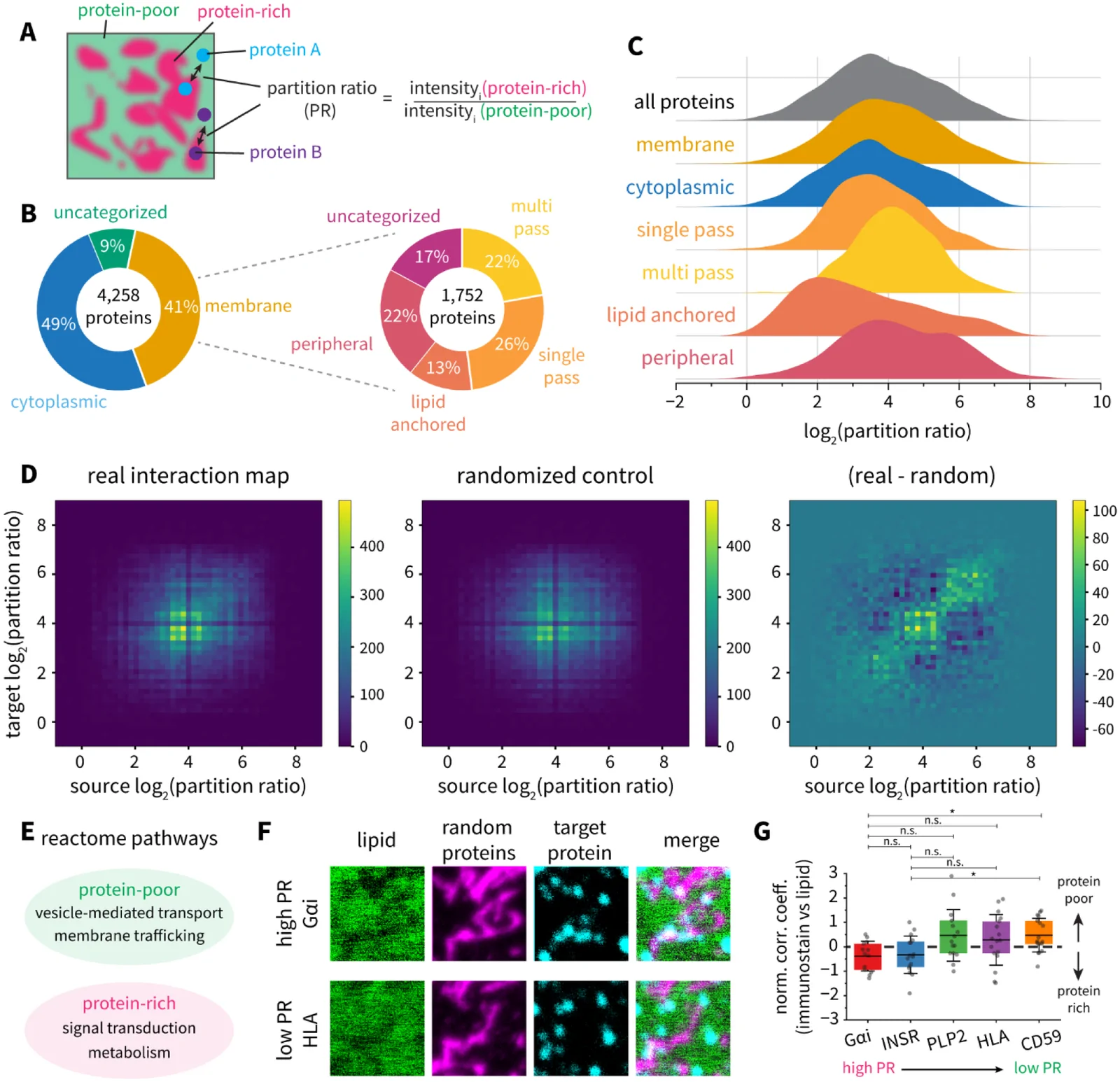
Protein content of protein-rich and protein-poor domains. (**A**) Schematic of the concept of protein partitioning between protein-rich and protein-poor domains. The partition ratio (PR) is defined as the ratio of intensities of a given protein in the protein-rich and the protein-poor vesicles. (**B**) Summary of the protein mass spectrometry data, showing the number and types of proteins characterized. About half of the total proteins are membrane proteins. 9% were uncategorized as either membrane proteins or cytoplasmic proteins based on their UniProt identifiers. The 17% of uncategorized membrane proteins were not annotated according to membrane protein type. (**C**) Distributions of the partition ratio for all proteins and different subsets of proteins in the data set. (**D**) Interactions between proteins in the data set. Pairs of proteins (source and target) known to interact with each other were identified using STRING, and their partition ratios are plotted. Heatmaps of the interaction map are shown for the real data (left), a randomized control (middle), and the difference between the real data and the randomized control (right). The difference heatmap is linear, indicating that a subset of the proteins in the data set (approximately a third) are known to interact with other proteins of similar partition ratios. (**E**) Reactome pathways enriched in the protein-poor and protein-rich domains, obtained from the subsets of proteins with the lowest (protein-poor) and highest (protein-rich) PR values, respectively. (**F**) Validation of the protein data set by immunostaining proteins with different PR values in unroofed cell membranes. Representative images (2 μm x 2 μm) of immunostaining against a protein with high PR (Gαi, top row) and a protein with low PR (HLA, bottom row). Lipid signal is shown in green, the nonspecific protein label (Cys-reactive) is shown in magenta, and the immunolabel against the specific protein is shown in cyan. (**G**) Summary of the immunolabeling data for five proteins, ranked by their PR values. For each protein, the normalized correlation coefficient between the lipid signal (which is depleted in protein-rich domains) and the specific immunolabel is shown. The normalization involved dividing by the absolute value of the correlation coefficient between the lipid and the protein signal, which was −0.1889 on average. The two proteins with higher PR have an anticorrelation coefficient that is about half of the lipid–protein anticorrelation in strength, while the proteins with lower PR show a weak positive correlation with the lipid signal. The box represents the middle 50% of the data (25–75% percentile range) and the error bar is the standard deviation. The statistical test used was Welch’s *t* test, corrected for multiple tests using the Holm method.

The distribution of partition ratios for different subsets of protein types is given in Figure 5C. Individual proteins are enriched to different extents in the protein-rich domains – some proteins are favored more than others. We point out that there are at least two reasons why a particular protein might have a high PR value: 1) the protein partitions favorably into protein-rich domains, or 2) the protein associates with another protein that partitions strongly into protein-rich domains. The PR distributions for most classes of proteins are similar – covering the whole range of PR values – but multipass membrane proteins tend to have higher PR values while lipid-anchored proteins (both GPI-linked and lipid tail anchored) have lower PR values on average (Figure 5C). We will return to this in the Discussion, when considering what mechanism underlies the formation of these protein-rich domains.

What role might protein sorting into these domains play? We wondered whether proteins with similar partition ratios were known to interact with each other. To test this possibility, we identified all known unique interacting pairs of proteins (from the set of 4,258 proteins) using STRING functional network analysis,^36^ and plotted a symmetrized (i.e., for each unique source and target, there are two points that are symmetric about the diagonal) heatmap of their partition ratios (Figure 5D – left panel). 2,721 of the 4,258 proteins had at least one interaction partner using the criteria used. If all interactions occurred between proteins of similar PR, we would expect a diagonal line. As a control, we randomized the pairing between the interactors and plotted the corresponding symmetrized heatmap on the same scale (Figure 5D – middle panel). To see if there is a correlation between PR and function, we subtracted the randomized data from the real interaction map (Figure 5D – right panel). What emerges is a clear linear trend – in other words, proteins that have similar PR values have been documented to interact as part of functional networks in living cells. This linear trend is accentuated for membrane proteins compared to cytoplasmic proteins in the data set (SI Appendix, Figure S8). With the caveat that STRING interactions do not necessitate spatial colocalization in the membrane, we suggest that organization into protein-rich and protein-poor domains could permit the spatial segregation of biological functions within the plasma membrane. Proteins with the highest PR values are involved in biological pathways such as signal transduction and metabolism, while proteins with the lowest PR values participate in vesicle-mediated transport and membrane trafficking (Figure 5E; SI Appendix, Figure S9).

Finally, to validate the measured protein partition ratios, we performed immunofluorescence experiments on five membrane proteins spanning the range of PR values to map them back onto protein-rich and protein-poor domains at the plasma membrane. We labeled unroofed membranes using a lipid label, a cysteine-reactive indiscriminate protein label, and an antibody against one of the five targets (Figure 5F). Representative images for one protein (Gαi) with a high PR and one protein with a lower PR (HLA) are shown in Figure 5F (other examples in SI Appendix, Figure S10). As we saw before, the indiscriminate protein labeling is anticorrelated with the lipid stain in both cases. However, most of the Gαi signal appears at or near these lipid shadows, which correspond to the protein-rich domains, while the HLA signal appears randomly distributed across the membrane (Figure 5F). We quantified this phenomenon for the five targets by calculating the correlation between the lipid stain and the immunostain for the protein (Figure 5G). Due to the low signal-to-noise, we normalized the correlation coefficient by the average correlation coefficient between lipid and protein (−0.1889; SI Appendix, Figure S10A) which we know is negative (Figure 1D–E). The two proteins with higher PR have an anticorrelation coefficient that is about half of the lipid–protein anticorrelation in strength, while the proteins with lower PR show a weak positive correlation with the lipid signal. In summary, these immunostaining experiments corroborate the partition ratios from the proteomics data and the connection between our studies of unroofed membranes and the cell-derived vesicles.

## Discussion

In the first part of this study we find that proteins in the plasma membrane of HEK cells are not randomly distributed but instead seem to exist in protein-rich domains on the order of 100 nm diameter, sometimes larger, separated by protein-poor regions (Figure 6). While past studies have documented the heterogeneous nature of cell membranes,^11–16^ none have isolated domains to characterize their protein and lipid composition. That we have isolated protein-rich and protein-poor domains in this study hinges on the hypothesis that protein-rich and protein-poor vesicles derive from protein-rich and protein-poor domains. The observation that membrane heterogeneity persists at least partially in GPMVs, which are the intermediate vesicles between the intact cell membrane and the SPMVs, lends support for this hypothesis (Figure 2D–F). The demonstration that high and low PR proteins are correlated to protein-rich and protein-poor domains, respectively, measured in unroofed membrane sheets, also supports the hypothesis (Figure 5F, G). In the discussion below we therefore use “vesicle” (the objects we isolated) and “domain” (the objects from which the vesicles come from) interchangeably, with the understanding that this remains a hypothesis. For instance, domains could conceivably reorganize during vesicle formation or downstream biochemical processing.

**Figure 6:**
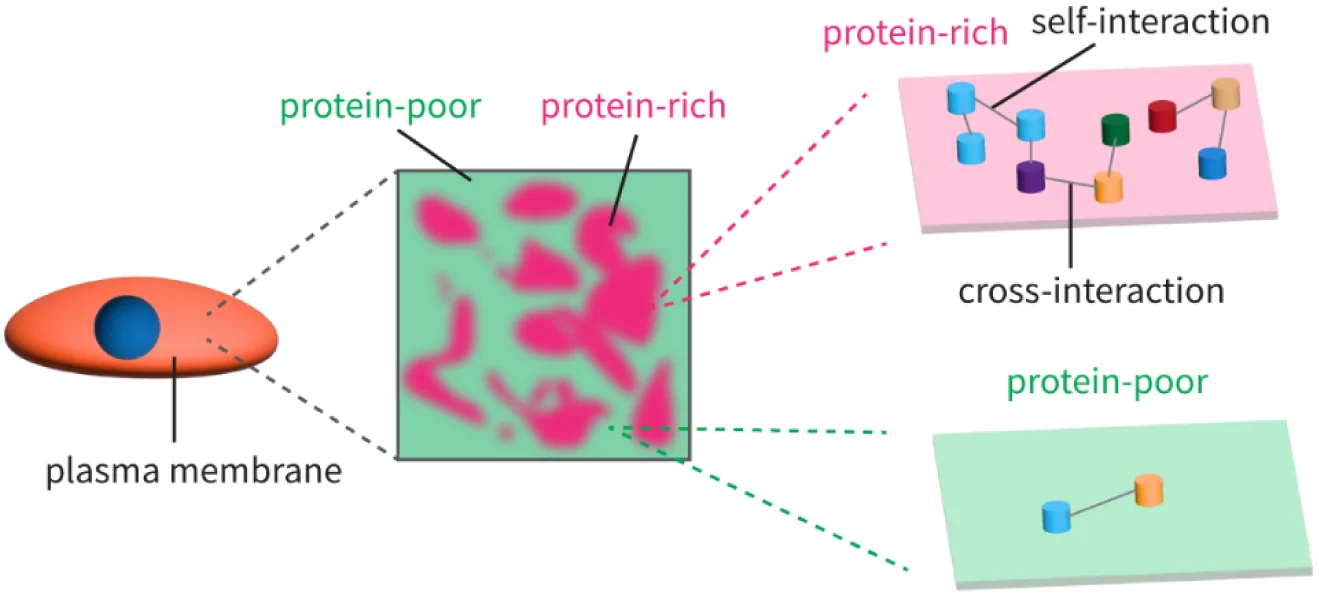
Schematic summarizing the protein-rich and protein-poor domains. The plasma membrane of living cells is organized into regions that are enriched in proteins, termed protein-rich domains, and regions that are poor in protein, called protein-poor domains.

Two major technical concerns tend to produce reflexive criticism of any attempts to interpret membrane organization. First, chemical fixation either due to incomplete immobilization or cross-linking effects can create artifacts. We note that others have demonstrated in live cells the existence of protein-rich domains similar to those we describe.^12^ Furthermore, while our data do not directly address the presence of these domains in living cells, they show that the domains are present in vesicles derived from cells that are not fixed. Second, the surface on which cells grow can influence membrane organization. But domains similar to those we describe have been observed in fixed cells grown on different substrates^11^ as well as in live cells.^12^ There is no doubt that proteins in contact with a surface can be immobilized, and that surfaces can and do influence membrane organization, but this is reasonable – after all, free floating cells are scarce in our bodies. Moreover, to invoke surface interactions as the underlying cause of the organization we observe would necessitate patterning on the surface to direct the organization.

### Do Protein-Rich Domains Exist in Different Types of Living Cells?

Protein-rich domains have been observed primarily in studies imaging the distributions of specific proteins in a variety of different cell types grown on different surfaces.^11–16^ When looking at the distribution of specific proteins on the cell membrane using negative stain transmission electron microscopy, one finds that antibody labels against the protein are predominantly found at high-contrast, dark-appearing regions of the cell membrane.^10,11,14,15,37^ These results have been supported by atomic force microscopy measurements^16^ and fluorescence microscopy of these domains,^12,16,17^ including in living cells. The presence and ubiquity of these domains, at least in the cell membranes of higher forms of life, seems likely. The existence of these domains thus modifies the picture of dilute, freely diffusing proteins in a sea of lipids, first painted by Singer and Nicolson in the 1970s,^38^ and adds to models of plasma membrane compartmentalization introduced since then.^1^

The dearth of studies discussing protein-rich domains and the anticorrelation we observed between the lipid and protein signal is surprising to us, but closer inspection of many other studies shows that these domains are likely present in work by others but have just been missed. The anticorrelation has been mentioned in at least one study before,^12^ but is also visible in data reported by others: for instance in studies of GPCR domains at the plasma membrane,^6^ and of B cell receptor clusters in live cells.^7^ Finally, aside from the imaging experiments, it has long been recognized that the plasma membrane can be biochemically isolated as multiple different subfractions using density centrifugation.^39^ In fact, it has been shown before that simply homogenizing plasma membrane fractions of a given density can give rise to fractions of higher and lower density, corresponding to protein-rich and protein-poor membranes compared to the input membranes.^40,41^ Our observations should thus be pertinent to most studies of how proteins are distributed at the cell membrane, both in imaging experiments^42^ and in single-particle tracking studies.^43^

### Red Blood Cells: A Model System for Plasma Membrane Organization?

Red blood cells have long been used as a model system for the plasma membrane because these cells lack internal membranes – thus removing the requirement to isolate the outer membrane from the more abundant inner membranes. Freeze fracture studies of intramembrane proteins in red blood cell membranes show homogeneously and densely packed proteins that are prone to aggregation upon treatment with trypsin or chemical agents.^44^ In contrast to such studies of red blood cells, freeze fracture experiments on the somata of neurons^45^ show much sparser and more clustered intramembrane particles that align more closely with our experiments. The cholesterol levels we measure in our cell-derived vesicles are much lower than in red blood cells (Figure 4C). The “textbook” number of ∼40% cholesterol in the plasma membrane derives largely from studies on red blood cells;^33^ immortalized cell lines have approximately 22% cholesterol in their plasma membranes.^46^ In summary, we think that red blood cells are quite specialized in their function and may not always be representative of most other cell types.

### Implications for Membrane-Dependent Biological Processes

We have recently shown that many membrane proteins appear to self-assemble into homotypic – and in some cases heterotypic – small clusters in the cell membrane called higher-order transient structures (HOTS).^10,47^ HOTS are formed by weak yet specific protein–protein interactions, which promote self-assembly between monomers diffusing in the cell membrane, and may increase the efficiency of signal transduction by locally concentrating molecules that function together, for instance, in a signaling pathway.^47^ It is notable that HOTS are concentrated inside protein-rich domains,^10^ as illustrated in Figure 6. Furthermore, others have shown that different kinds of proteins are nonrandomly distributed inside protein-rich domains.^12^ In the present study we find that functionally linked proteins are correlated to their PR, which would be expected if functionally linked proteins are localized spatially (Figure 5D). Taken together, these findings make plausible the idea that protein rich domains in cell membranes represent one of multiple levels of molecular organization in cell membranes. The protein rich domains represent relatively larger collections of different proteins, which then spontaneously self-organize inside the protein rich domains through other mechanisms such as specific self-assembly that underlies HOTS formation. This idea, if correct, naturally raises the question, what drives the formation of the protein rich domains in the first place?

### What Is the Mechanism of Assembly of These Protein-Rich Domains?

Protein–protein interactions, lipid rafts, and cytoskeleton-mediated corrals have all been proposed to contribute to plasma membrane organization. Here we find that isolated plasma membrane-derived vesicles, lacking cytoskeletal organizations, contain protein-rich and protein-poor domains. Protein-rich domains thus seem to form spontaneously from the protein–lipid mixture in the cell membrane. The cytoskeleton does likely still contribute to controlling the formation and size of these domains in cell membranes – for instance, it has been shown that disruption of cytoskeletal elements leads to larger clusters of many proteins.^11,48^ Perhaps the cytoskeletal mesh acts as a diffusion barrier, not just to confine proteins and lipids in certain zones, but also to prevent larger self-assemblies of proteins.

We found that protein-rich vesicles are rich in cholesterol and sphingolipids, thus, correlating protein density to these ordered lipids. However, we are unsure which came first. Do coalescing protein clusters bring these ordered lipids with them, or do the lipids form domains into which the proteins partition? The original observations surrounding the lipid raft model – that some proteins are resistant to detergent extraction – could be explained by either mechanism.^49,50^ We favor the idea that most membrane proteins (except some types of lipid-anchored proteins that are more akin to lipids) are inherently lipophobic to some extent, in that membrane proteins tend to associate in membranes to exclude the solvent (lipids), and that perhaps this process produces some form of phase separation.^51^ Analogous to protein folding in water,^52^ perhaps this process is driven by the entropy gained from lipids released from the annular shell upon protein association. The reason we favor a mechanism along the lines of this later picture is simply that most membrane proteins do not partition into liquid ordered (raft) phases when in the presence of liquid disordered (nonraft) phases (see SI Appendix, Figure S11 and^53,54^ for examples).

It remains to be seen what precise mechanisms segregate the cell membrane into protein-rich and protein-poor domains. There must be identifiable molecular properties of proteins and/or lipids that drives this process. We deposit the lists of proteins and lipids enriched in protein-rich domains to promote further analysis to uncover the mechanism.

## Conclusion

In summary, the data here point to the existence of protein-rich and protein-poor domains in cell membranes and demonstrate how to isolate and characterize them. We conclude that most, but not all membrane proteins are concentrated in these protein-rich domains. Proteins that are functionally connected reside within these domains. Ordered lipids such as sphingomyelin and cholesterol are also enriched in these domains. Finally, the existence of these domains in the absence of cytoskeletal elements points to spontaneous assembly of components underlying their formation.

## Materials and Methods

### Cell Culture

HEK293S GnTI^–^ cells were used in both adherent and suspension culture. Suspension cells to produce cell-derived vesicles were cultured in Freestyle 293 medium (GIBCO) supplemented with 2% fetal bovine serum, 100 U/mL penicillin, and 100 U/mL streptomycin at 37 °C in 8% CO_2_. Adherent cells for imaging experiments were cultured in Dulbecco’s Modified Eagle Medium (GIBCO) supplemented with 10% fetal bovine serum and 2 mM l-glutamine.

### Preparation of Intact and Unroofed Cells for Imaging

Adherent HEK293S GnTI^–^ cells were grown either on high-performance glass coverslips (Zeiss, 0.17 mm thick) coated with poly-d-lysine (PDL) or on glass coverslips coated with both PDL and laminin (Corning BioCoat). For most experiments, cells were unroofed using a stream of Dulbecco’s phosphate-buffered saline (DPBS) through a 1 mL pipet applied at a ∼45° angle (∼10–15 applications per coverslip). Sonication (Qsonica Q125 sonicator equipped with a CL-18 probe) at a distance of ∼1 cm from the coverslip with 20% amplitude for ∼1 s was also used to ensure similar results as with shear flow.

Indiscriminate labeling of proteins was carried out by adding 15 μM of fluorophore-maleimide conjugate (e.g., STAR RED-maleimide) in DMSO and incubating for ∼20 min at room temperature. Lipid labeling was accomplished by incorporating the CellMask family of probes (Thermo Fisher) at dilutions of 1:1000 to 1:4000, or by adding 1 μg/mL of AF594-PE (Avanti Research) in DMSO, or by adding 250 ng/mL of STAR RED-PEG2000-DPPE (custom synthesis from Abberior) in DMSO. Typical lipid label incubation times were 5–20 min. Membranes were washed three times using DPBS to remove excess stain, fixed using 4% paraformaldehyde (PFA), washed again with DPBS three times, and then mounted on glass slides as detailed below.

The intact cell labeling was performed by incubating cells grown on the coated coverslips with STAR 580-maleimide (15 μM) and STAR RED-PEG2000-DPPE (250 ng/mL) for 5–10 min at room temperature. Cells were then immediately washed and fixed using 4% PFA for 10 min, washed again, and then mounted on glass slides.

Coverslips with fixed, intact cells, or with fixed, unroofed membranes were mounted on glass slides with ProLong Diamond Antifade Mountant (Thermo Fisher), sealed using nail polish, and allowed to cure for 1–2 days before imaging.

### Preparation of GPMVs and GUVs for Fluorescence Imaging

Giant plasma membrane vesicles (GPMVs) were prepared from adherent cells grown in 6-well plates. Cells were washed with GPMV buffer (10 mM Hepes pH 7.4, 140 mM NaCl, 10 mM KCl, 2 mM CaCl_2_) twice before adding 2 mL of GPMV buffer with 7.5 mM N-ethylmaleimide (NEM) and incubating for 1.5 h at 37 °C and 5% CO_2_. Two hundred microliters (200 μL) of the GPMV-containing solution was applied to a glass coverslip coated with PDL and laminin, let sit for 5 min, then GPMV buffer (without NEM) was added and the GPMVs were allowed to settle and burst for 1–2 h. GPMVs were then labeled using CellMask Orange (1:1000) for 20 min at room temperature, washed three times gently using NEM-free GPMV buffer, and then mounted on a glass slide as detailed before.

Giant unilamellar vesicles (GUVs) comprised of synthetic lipids were prepared by dehydration–rehydration. Lipids (POPC or a 3:1:1 POPC:POPE:POPG mixture) were mixed in chloroform and dried to a thin film on 35 mm glass-bottomed dishes (Maktek) using a stream of argon and a vacuum desiccator for a few hours. The lipid film was rehydrated using 100 μL of GUV rehydration buffer (10 mM Hepes pH 7.4, 50 mM KCl, 200 mM sucrose) and let sit overnight at 4 °C. The next day, the GUV-containing solution was harvested and applied to a PDL/laminin-coated coverslip, let sit for 5 min, and then GUV bath buffer (10 mM Hepes pH 7.4, ∼180 mM KCl, osmolarity matched) was added to the dish and the GUVs were allowed to settle and burst for 1–2 h. GUVs were labeled by incubating with CellMask Orange (1:1000) for 20 min at room temperature, washed three times with GUV bath buffer, and then mounted on a glass slide.

### Confocal/STED Microscopy

Confocal and STED images were collected on a Facility line STED microscope (Abberior Instruments). The microscope was equipped with an Olympus IX83 stand, Olympus UPLXAPO × 100/1.45 NA oil objective, pulsed excitation lasers with time gating (405, 488, 561, and 640 nm) and a 775 nm pulsed STED depletion laser, 4 Avalanche Photo Diode detectors, adaptive illumination packages (DyMIN/RESCue), and a deformable mirror for correction of spherical aberrations. The Abberior Imspector software was used for image acquisition. Fluorophore excitation was performed at 488 nm (AF488-derivatives or CellMask Green) 580 nm (AF594-derivatives, STAR 580-derivatives or CellMask Orange) and 640 nm (STAR RED-derivatives), whereas depletion for STAR 580 or STAR RED was achieved using a wavelength of 775 nm. A pixel size of 15 nm was used in 2D STED mode. Excitation laser power, depletion laser power, line averaging/accumulation, and pixel dwell time were optimized for each imaging session to balance the signal-to-noise ratio and the resolution of the STED images. Images were exported as OBF files and visualized and analyzed using Fiji/ImageJ.

### TIRF Microscopy

TIRF imaging was performed using a Nikon Eclipse Ti2-E inverted microscope equipped with a Tokai Hit stage-top incubator. Laser illumination was provided by a multimodal laser launch with wavelengths of 405, 488, 561, and 647 nm. The laser beam was directed through a Nikon CFI SR HP Apochromat TIRF 100× oil-immersion objective (NA 1.49, WD 0.15 mm). The microscope was equipped with a Gataca iLas 2 controller for azimuthal TIRF control. The angle of illumination was adjusted to achieve total internal reflection, and the penetration depth of the evanescent wave was calibrated to ∼70–100 nm. Fluorescence emission was collected after passing through the appropriate filter and captured by a Hamamatsu ORCA-Fusion BT camera. Images were acquired sequentially for each channel with typical exposure times of ∼100 ms and laser power was set at about 20% of the maximum. Finally, sample focus was maintained using the Nikon Perfect Focus System and the system was controlled using Nikon NIS-Elements.

### Image Visualization and Analysis

Images were visualized using Fiji/ImageJ.^55^ The histogram analysis was performed by drawing a ∼300 nm-sided square and reading the standard deviation from the Histogram tool. Multiple locations within each image were taken for both the background and the membrane, and visibly brighter regions (assumed to be vesicles or incompletely unroofed regions) or holes in the membrane were excluded. Pearson cross-correlation coefficients between different channels were calculated using the inbuilt plugin, using multiple squares of ∼2 μm × 2 μm within each image. Again, notably brighter regions and holes were excluded as these would bias the calculation.

### Production and Purification of Protein-Rich and Protein-Poor SPMVs

The protocol for production of SPMVs is very similar to that reported before.^18^ 1 L of HEK293S GnTI^–^ cells were grown to a density of ∼3 × 10^6^/mL. Cells were harvested by pelleting at 500 × g for 10 min. The supernatant was decanted and the cells were gently resuspended in 200 mL of GPMV buffer to remove residual media and secreted vesicles (10 mM Hepes pH 7.4, 140 mM NaCl, 10 mM KCl, 2 mM CaCl_2_). The cells were harvested again at 500 × g for 10 min and resuspended in ∼400 mL of GPMV buffer with 7.5 mM NEM. Around 100 mL of this suspension was transferred to each of four 250 mL flasks, and incubated at 37 °C and 8% CO2 for 1.5 h with shaking at 130 rpm (25 mm throw; Infors HT). The flasks were shaken by hand to dislodge any attached vesicles, and the suspension was spun at 3,000 × g for 10 min to remove cell debris and large vesicles.

Ten percent glycerol was added to the harvested vesicles and a probe sonicator (Branson 250, 1/2″ tip) was used to disrupt these vesicles at 40% amplitude for three 30 s pulses with ∼1 min chilling on ice in between pulses. The vesicles were then pelleted by ultracentrifugation at 38,000 rpm (SW 70 Ti rotor) at 4 °C for 40 min, and each pellet was resuspended by trituration in 1 mL of GPMV buffer with 10% glycerol and sonicated twice for 10 s in a bath sonicator (Branson M1800). Aggregates were removed by pelleting at 3,500 × g for 10 min and the vesicles were pooled and loaded onto sucrose density gradients (10%, 12%, 25%, 35%, 40%, and 50 wt %/vol sucrose steps) prepared in ultracentrifuge tubes. Around 3 mL of sample was loaded on top of each gradient and spun for 20–24 h at 38,000 rpm (∼170,000 × g; SW 41 Ti) and 4 °C. Fractions of ∼1 mL in size were collected from the gradients after the spin and were individually washed (to remove sucrose) and concentrated in 2 mL 100 kDa MWCO spin filters (Amicon) until a final volume of ∼50 μL for each fraction. These fractions were aliquoted, flash frozen using liquid nitrogen, and stored at −80 °C until use. Fraction number 1 and fraction number 6 from multiple experiments were used as the protein-poor and protein-rich fractions, respectively.

### Grid Preparation and Cryo-EM Imaging

Grids for cryo-electron microscopy (cryo-EM) were prepared as described before.^56^ Quantifoil R1.2/1.3 400 mesh holey carbon Au grids were glow-discharged for 22 s, and 3.5 μL of concentrated membrane vesicles were applied and incubated for 3–5 min at 20 °C with a humidity of 100%. The grids were blotted manually from the bottom edge of the grid with a piece of filter paper. Another 3.5 μL of sample was applied, and the grids were blotted using a Vitrobot Mark IV with a blot force of 0 and blot time of 3 s after 20 s of incubation. The grids were then flash-frozen in liquid ethane and stored in liquid nitrogen until data collection.

Cryo-EM images were collected on an FEI Talos Arctica transmission electron microscope operating at 200 kV. The microscope was equipped with an autoloader and a Gatan K2 camera. A target defocus of −3.5 μm was used to acquire the images.

### Protein and Lipid Quantification

Total protein content was quantified using a plate reader-based BCA assay (Thermo Fisher). The manufacturer’s protocol was used and 2% SDS was added to all the samples prior to the assay. Albumin was used to establish a linear calibration between 20 μg/mL and 2000 μg/mL. Protein content in the vesicle fractions was quantified using a plate reader (Tecan Infinite M1000) by measuring absorbance at 562 nm and using the calibration to calculate the protein concentration.

Lipid and cholesterol concentrations were measured using ^1^H solution NMR. A small amount of lipid vesicle fractions (∼10 μL) was dissolved in ∼550 μL of 2:1 (v/v) CD_3_OD:CDCl_3_ containing 100 μM TMSP as an internal standard and transferred to a 5 mm NMR tube for measurement. One dimensional ^1^H spectra were acquired using typically ∼32 transients on a 600 MHz (^1^H Larmor frequency) Bruker instrument equipped with an Avance Neo console and a 5 mm HCN cryoprobe. Peak integration was carried out in Topspin after phasing and baseline correction and care was taken to avoid overlapping regions for quantification.

### Red Blood Cell Lipid Extraction

Extraction of total lipids from red blood cells (RBCs) was carried out using the Bligh–Dyer method.^57^ Red blood cells (Innovative Research) were washed three times with DPBS by pelleting at 3,000 × g for 5 min. Two preparations were tested that gave very similar results. First, washed RBCs were directly used for extraction by incubating 1 part of RBC slurry (∼80% water) with 2 parts of methanol and 1 part chloroform for 1 h with agitation. Then 1 part of water and 1 part of chloroform were added, the mixture was filtered and allowed to phase separate and the bottom organic phase containing lipids was collected. The organic solvent was evaporated using a stream of argon gas and deuterated NMR solvent was added to the dried lipids to solubilize them. NMR measurements were carried out as detailed before. In the second method, RBC ghosts were first prepared by two rounds of hypo-osmotic shock (5 mM potassium phosphate buffer at pH 7.4, 0.2 mM EDTA, and 1 mM MgCl_2_) followed by four washes and pelleting,^58^ and then the lipids were extracted for NMR measurements as described for the first method.

### LC/MS Lipidomics

LC-MS grade acetonitrile (ACN), isopropanol (IPA), methanol (MeOH), and 1-butanol (BuOH) were purchased from Fisher Scientific. High purity deionized water was filtered from Millipore (18 OMG). Lipid standards were purchased from Avanti Polar Lipids (Alabaster, AL, USA). Ammonium formate was obtained from Sigma-Aldrich in the best available grade.

Vesicles were extracted by adding 50:50 MeOH:BuOH + 10 mM ammonium formate at a 20:1 solvent-to-sample volume ratio. The vesicle–solvent mixture was subjected to bead-beating for 45 s using a Tissuelyser cell disrupter (Qiagen), followed by 15 min sonication at 20 °C. The mixture was centrifuged for 10 min at maximum speed. The above procedures were repeated twice to pool the supernatant. The final lipid extracts were dried in a Vacufuge (Eppendorf) and stored at −80 °C until analysis. On the day of lipidomics sample run, 40 μL of 50:50 MeOH:BuOH + 10 mM ammonium formate was added to the dry-down lipids, vortexed and sonicated to solubilize the lipids. The supernatant was transferred to sample vials for positive and negative ion lipidomics data acquisition.

The LC/MS platform for lipidomics analysis was as described previously,^59^ consisting of an Agilent Model 1290 Infinity II liquid chromatography system coupled to an Agilent 6550 iFunnel time-of-flight MS analyzer. An Agilent ZORBAX Eclipse Plus C18, 100 × 2.1 mm, 1.8 μm (Cat# 959758-902) reversed phase column was used for the separation. Mobile phases were (A) 10 mM ammonium formate with 5 μM Agilent deactivator additive (Cat# 5191-3940) in 5:3:2 water:ACN:IPA and (B) 10 mM ammonium formate in 1:9:90 water:ACN:IPA. Column temperature was set at 55 °C and autosampler temperature was at 20 °C. The flow rate was 0.4 mL/min with sample injection volume of 4 μL. The following gradient was applied: 0 min, 15% B; 0–2.5 min, to 50% B; 2.5–2.6 min, to 57% B, 2.6–9 min, to 70% B; 9–9.1 min, to 93% B; 9.1–11.1 min, to 96% B; 11.1–15 min, 100% B; 15–20 min, 15% B.

Raw data were analyzed using MassHunter Qualitative Analysis (10.0), Mass Profinder 10.0 and MassProfiler Professional (MPP) 15.1 software (Agilent Technologies). Lipid peak chromatograms were extracted against an in-house database created using MassHunter PCDL Manager 8.0 (Agilent Technologies) and lipid standards, based on monoisotopic mass (<5 ppm mass accuracy) and chromatographic retention time matches (<0.5 min). Matched lipids were further confirmed by comparing MS/MS fragmentation pattern to the corresponding lipid species standards.

Peak area ratios of extracted lipids were used to compare differences in partitioning into protein-rich and protein-poor vesicles, after normalizing each sample for the total integrated area of all identified lipids (to account for different amounts of lipids injected). The partition ratio for each lipid species was calculated as the pairwise ratio of the intensity of that lipid in the protein-rich vesicles divided by the intensity of the same species in the protein-poor vesicles.

### Protein Mass Spectrometry

SPMVs were diluted to afford solutions containing 0.8 μg of protein in 30 μL PBS (as measured by BCA), then sonicated for 5 min in a water bath. Three μL of a stock of dithiothreitol (100 mM in water) was added to each sample, and the samples heated to 95 °C for 5 min. Three μL of a stock of iodoacetamide (200 mM in water) was then added to each sample and the mixtures incubated at room temperature in the dark. The SP3 protocol was then used to perform protein-level cleanup.^60^ Samples were then again sonicated for 5 min in a water bath. 7.5 μL of Sera-Mag SpeedBeads (a 1:1 ratio of GE cat. No. 45152105050250 and cat. No. 65152105050250, washed three times with water and suspended at a density of 50 mg/mL) was added to each sample, and 36 μL of acetonitrile (UHPLC-MS grade) added. These mixtures were then allowed to stand without agitation for 8 min at room temperature. A magnetic rack was used to pellet the beads, and the supernatant aspirated. Beads were then washed twice with 100 μL of UHPLC-MS grade 80% ethanol/water and twice with UHPLC-MS grade acetonitrile, each time with resuspension. Beads were then resuspended in 50 μL of ammonium bicarbonate in water containing 0.2 μg MS-grade trypsin (Pierce, cat. No. 90058, prepared from dilution from a 1 mg/mL stock solution in 50 mM acetic acid) and sonicated in a water bath for 30 s. The suspended beads were then incubated at 37 °C for 16 h with continuous inversion. Samples were then centrifuged (21,000 × g, 10 min) and the supernatant filtered using cellulose acetate cartridges (Corning Costar Spin-X Centrifuge Tube Filters, 0.22 μm pore CA membrane, PN 8161). The flowthrough was then desalted using Pierce C18 spin tips according to the manufacturer protocol with formic acid substituting trifluoroacetic acid, and dried peptide samples resuspended in 20 μL 2% UHPLC-MS grade acetonitrile/water containing 0.1% formic acid.

Data was obtained using a timsTOF Pro 2 mass spectrometer system integrated with a nanoElute UHPLC. A Waters nanoEase Symmetry C18 trap column (cat. No. 186008821, 5 μm particle size, 180 μm ID x 20 mm length) was used for online sample cleanup with an IonOpticks Aurora Ultimate column (75 μm ID, 1.5 μm particle size, 25 cm length) with integrated captivespray emitter used for analysis. A two-column separation method was used with a 60 min gradient spanning 2–37% acetonitrile/water at a 150 nL/min flow rate, with the default long gradient DIA method used for MS data acquisition (1 s cycle time). Raw data was processed using DIANN using an in-silico spectral library generated from a human proteome to provide protein-level IDs using default settings.^61^

Raw intensities obtained for each protein were corrected within each sample using VSN normalization in R.^34^ The normalized intensities were then corrected by the known protein and lipid concentrations for each sample to adjust for the area of the cell membrane. The partition ratio (PR) for each protein was calculated as the pairwise ratio of the intensity of that protein in the protein-rich vesicles divided by the intensity of the same protein in the protein-poor vesicles. We only report results for proteins that were detected in at least two independent samples for each set of vesicles. For each protein with a PR, we mined the UniProt database^35^ to obtain annotations for protein subcellular localization and for basic structural information.

To query proteins known to interact, we used the STRING protein–protein interaction database^36^ running on the Cytoscape StringApp.^62^ The 4,258 proteins that were identified from the mass spectrometry data were input into the STRING database and a cutoff of 0.8 was used to filter out less certain interactions. The output from STRING is a list of all unique (meaning each pair only shows up once, not twice) pairwise interactions between the proteins. 2,721 of the 4,258 proteins had at least one interactor that passed the cutoff filter. A Python script was used to obtain the PR values for all of the pairwise interactors identified from STRING, symmetrize the output (i.e., for each pair there are now two points that are symmetric about the diagonal), and then plot the symmetrized data as a heatmap with a defined number and width of bins and scales.

### Immunofluorescence Experiments in Unroofed Membranes

Primary antibodies against each target were purchased and screened for nonspecific labeling and optimal labeling dilutions before use. The following primary antibodies were used; Gαi: Thermo MA5-12800, INSR: Abcam ab245688, PLP2: Abcam ab208266, HLA-ABC: Thermo MA5-11723, and CD59: Thermo MA1-19133. AF488-labeled secondary antibodies against the appropriate host were used.

Cells were plated on PDL/laminin-coated coverslips. Residual media was removed by washing twice with DPBS. The cells were then unroofed by shear flow and then washed three times with DPBS. Indiscriminate protein and lipid labeling was carried out using STAR RED-maleimide and CellMask Orange, respectively, as detailed before. The unroofed membranes were washed three times, blocked with 2% bovine serum albumin (BSA) in DPBS for 30 min, washed again two times, and then labeled with primary antibody for 45 min at the optimal dilution for each antibody (in the presence of 2% BSA). Excess primary antibody was removed by washing three times and the secondary antibody was then added at a dilution of 1:500 with 2% BSA for 45 min. Excess secondary antibody was removed by washing three times and the samples were fixed with 4% PFA, washed again, and then mounted on a glass slide for imaging.

### Preparation of Uniform and Phase-Separated GUVs Containing Proteins

GIRK, Plp2, CD63, and Gβγ were expressed and purified as described before^63–65^ and reconstituted into small unilamellar vesicles (SUVs) of the appropriate lipid composition (soy PC, 2:2:1 or 1:2:2 DOPC:egg SM:cholesterol, all from Avanti Polar Lipids) as detailed before.^66^ 0.1%–0.4% Rhodamine-PE (18:1) or Ganglioside GM1 (ovine brain) were doped in as fiducials for the liquid disordered and liquid ordered phases, respectively, as necessary. GUVs were generated by electroformation^64,67^ using indium tin oxide glass slides in an incubator oven set at ∼50 °C. GM1-labeling was performed by addition of AF488-cholera toxin B (Thermo) after GUVs were made. GUVs were imaged on 35 mm collagen-coated glass-bottomed dishes (Maktek) using an ECHO Revolve microscope equipped with a 100× oil objective.

## Supplementary Material





## Data Availability

Partition ratios from the proteomics and lipidomics analysis are deposited with the manuscript. All other data are available from the authors upon request.

## Notes

List of proteins and lipids identified by mass spectrometry and their partition ratios (XLSX)
Additional fluorescence microscopy data, functional network analysis, lipid MS analysis, and cryo-EM images (PDF)

## §

Department of Biochemistry and Molecular Biology, University of Iowa, Iowa City, IA 52242, USA

## ∥

Department of Biochemistry and Molecular Biology, University of Florida, Gainesville, FL 32610, USA.

## ⊥

Steven Gross deceased on September 1, 2025.

## References

[1] Laude A. J., Prior I. A. (2004). Plasma Membrane Microdomains: Organization, Function
and Trafficking (Review). Mol. Membr. Biol..

[2] Bernardino
de la Serna J., Schütz G. J., Eggeling C., Cebecauer M. (2016). There Is No
Simple Model of the Plasma Membrane Organization. Front. Cell Dev. Biol..

[3] Garcia-Parajo M. F., Cambi A., Torreno-Pina J. A., Thompson N., Jacobson K. (2014). Nanoclustering
as a Dominant Feature of Plasma Membrane Organization. J. Cell Sci..

[4] Sieber J. J., Willig K. I., Kutzner C., Gerding-Reimers C., Harke B., Donnert G., Rammner B., Eggeling C., Hell S. W., Grubmüller H., Lang T. (2007). Anatomy and Dynamics
of a Supramolecular Membrane Protein Cluster. Science.

[5] Feng W., Zhang M. (2009). Organization and Dynamics of PDZ-Domain-Related Supramodules in the
Postsynaptic Density. Nat. Rev. Neurosci..

[6] Kockelkoren G., Lauritsen L., Shuttle C. G., Kazepidou E., Vonkova I., Zhang Y., Breuer A., Kennard C., Brunetti R. M., D’Este E., Weiner O. D., Uline M., Stamou D. (2024). Molecular Mechanism
of GPCR Spatial Organization at
the Plasma Membrane. Nat. Chem. Biol..

[7] Shelby S. A., Castello-Serrano I., Wisser K. C., Levental I., Veatch S. L. (2023). Membrane
Phase Separation Drives Responsive Assembly of Receptor Signaling
Domains. Nat. Chem. Biol..

[8] Scarselli M., Annibale P., Radenovic A. (2012). Cell Type-Specific
Β2-Adrenergic
Receptor Clusters Identified Using Photoactivated Localization Microscopy
Are Not Lipid Raft Related, but Depend on Actin Cytoskeleton Integrity
*. J. Biol. Chem..

[9] Kusumi A., Fujiwara T. K., Chadda R., Xie M., Tsunoyama T. A., Kalay Z., Kasai R. S., Suzuki K. G. N. (2012). Dynamic
Organizing
Principles of the Plasma Membrane That Regulate Signal Transduction:
Commemorating the Fortieth Anniversary of Singer and Nicolson’s
Fluid-Mosaic Model. Annu. Rev. Cell Dev. Biol..

[10] Zhang Y., Mazal H., Mandala V. S., Pérez-Mitta G., Sondoghdar V., Haselwandter C. A., MacKinnon R. (2025). Higher-Order
Transient Membrane Protein Structures. Proc.
Natl. Acad. Sci. U. S. A..

[11] Lillemeier B. F., Pfeiffer J. R., Surviladze Z., Wilson B. S., Davis M. M. (2006). Plasma
Membrane-Associated Proteins Are Clustered into Islands Attached to
the Cytoskeleton. Proc. Natl. Acad. Sci. U.
S. A..

[12] Saka S. K., Honigmann A., Eggeling C., Hell S. W., Lang T., Rizzoli S. O. (2014). Multi-Protein Assemblies Underlie the Mesoscale Organization
of the Plasma Membrane. Nat. Commun..

[13] Nicolson G. L., Hyman R., Singer S. J. (1971). The Two-Dimensional
Topographic Distribution
of H-2 Histocompatibility Alloantigens on Mouse Red Blood Cell Membranes. J. Cell Biol..

[14] Wilson B. S., Pfeiffer J. R., Oliver J. M. (2000). Observing
Fcεri Signaling from
the Inside of the Mast Cell Membrane. J. Cell
Biol..

[15] Wilson B. S., Pfeiffer J. R., Surviladze Z., Gaudet E. A., Oliver J. M. (2001). High Resolution
Mapping of Mast Cell Membranes Reveals Primary and Secondary Domains
of FcεRI and LAT. J. Cell Biol..

[16] Frankel D. J., Pfeiffer J. R., Surviladze Z., Johnson A. E., Oliver J. M., Wilson B. S., Burns A. R. (2006). Revealing the Topography of Cellular
Membrane Domains by Combined Atomic Force Microscopy/Fluorescence
Imaging. Biophys. J..

[17] Spira F., Mueller N. S., Beck G., von Olshausen P., Beig J., Wedlich-Söldner R. (2012). Patchwork
Organization
of the Yeast Plasma Membrane into Numerous Coexisting Domains. Nat. Cell Biol..

[18] Tao X., Zhao C., MacKinnon R. (2023). Membrane Protein
Isolation and Structure
Determination in Cell-Derived Membrane Vesicles. Proc. Natl. Acad. Sci. U. S. A..

[19] Morone N., Usukura E., Narita A., Usukura J. (2020). Improved Unroofing
Protocols for Cryo-Electron Microscopy, Atomic Force Microscopy and
Freeze-Etching Electron Microscopy and the Associated Mechanisms. Microscopy.

[20] Hell S. W., Wichmann J. (1994). Breaking the Diffraction Resolution Limit by Stimulated
Emission: Stimulated-Emission-Depletion Fluorescence Microscopy. Opt. Lett..

[21] Sezgin E. (2025). Plasma Membrane
Labelling Efficiency, Internalization and Partitioning of Functionalized
Fluorescent Lipids as a Function of Lipid Structure. RSC Chem. Biol.

[22] Parmryd I., Önfelt B. (2013). Consequences
of Membrane Topography. FEBS J..

[23] van
Rheenen J., Jalink K. (2002). Agonist-Induced PIP2 Hydrolysis Inhibits
Cortical Actin Dynamics: Regulation at a Global but Not at a Micrometer
Scale. Mol. Biol. Cell.

[24] Scott R. E. (1976). Plasma
Membrane Vesiculation: A New Technique for Isolation of Plasma Membranes. Science.

[25] Levental I., Grzybek M., Simons K. (2011). Raft Domains
of Variable Properties
and Compositions in Plasma Membrane Vesicles. Proc. Natl. Acad. Sci. U. S. A..

[26] Holowka D., Baird B. (1983). Structural Studies
on the Membrane-Bound Immunoglobulin E-Receptor
Complex. 1. Characterization of Large Plasma Membrane Vesicles from
Rat Basophilic Leukemia Cells and Insertion of Amphipathic Fluorescent
Probes. Biochemistry.

[27] Brakke M. K. (1951). Density
Gradient Centrifugation: A New Separation Technique1. J. Am. Chem. Soc..

[28] Smith P. K., Krohn R. I., Hermanson G. T., Mallia A. K., Gartner F. H., Provenzano M. D., Fujimoto E. K., Goeke N. M., Olson B. J., Klenk D. C. (1985). Measurement
of Protein Using Bicinchoninic Acid. Anal. Biochem..

[29] Sparling M. L., Zidovetzki R., Muller L., Chan S. I. (1989). Analysis of Membrane
Lipids by 500 MHz 1H NMR. Anal. Biochem..

[30] Zwall R. F. A., Roelofsen B., Michael Colley C. (1973). Localization of Red Cell Membrane
Constituents. Biochim. Biophys. Acta BBA - Rev.
Biomembr..

[31] Dupuy A. D., Engelman D. M. (2008). Protein Area Occupancy at the Center
of the Red Blood
Cell Membrane. Proc. Natl. Acad. Sci. U. S.
A..

[32] Kremmer T., Wisher M. H., Evans W. H. (1976). The Lipid Composition of Plasma Membrane
Subfractions Originating from the Three Major Functional Domains of
the Rat Hepatocyte Cell Surface. Biochim. Biophys.
Acta, Biomembr..

[33] Dodge J. T., Phillips G. B. (1967). Composition of Phospholipids and
of Phospholipid Fatty
Acids and Aldehydes in Human Red Cells. J. Lipid
Res..

[34] Välikangas T., Suomi T., Elo L. L. (2018). A Systematic Evaluation
of Normalization
Methods in Quantitative Label-Free Proteomics. Brief Bioinform..

[35] The UniProt Consortium. UniProt: the Universal Protein Knowledgebase in 2025. Nucleic Acids Res. 2025, 53(D1), D609–D617. 10.1093/nar/gkae1010. 39552041 PMC11701636

[36] Szklarczyk D., Kirsch R., Koutrouli M., Nastou K., Mehryary F., Hachilif R., Gable A. L., Fang T., Doncheva N. T., Pyysalo S., Bork P., Jensen L. J., von Mering C. (2023). The STRING
Database in 2023: Protein–Protein Association Networks and
Functional Enrichment Analyses for Any Sequenced Genome of Interest. Nucleic Acids Res..

[37] Lillemeier B. F., Mörtelmaier M. A., Forstner M. B., Huppa J. B., Groves J. T., Davis M. M. (2010). TCR and
Lat Are Expressed on Separate
Protein Islands on T Cell Membranes and Concatenate during Activation. Nat. Immunol..

[38] Singer S. J., Nicolson G. L. (1972). The Fluid Mosaic
Model of the Structure of Cell Membranes. Science.

[39] DePierre J. W., Karnovsky M. L. (1973). Plasma Membranes of Mammalian Cells: A Review of Methods
for Their Characterization and Isolation. J.
Cell Biol..

[40] Wibo M., Thinès-Sempoux D., Amar-Costesec A., Beaufay H., Godelaine D. (1981). Analytical Study of Microsomes and
Isolated Subcellular Membranes from Rat Liver VIII. Subfractionation
of Preparations Enriched with Plasma Membranes, Outer Mitochondrial
Membranes, or Golgi Complex Membranes. J. Cell
Biol..

[41] Evans W. H. (1969). Subfractionation
of Rat Liver Plasma Membranes. FEBS Lett..

[42] Sato D., Hernández-Hernández G., Matsumoto C., Tajada S., Moreno C. M., Dixon R. E., O’Dwyer S., Navedo M. F., Trimmer J. S., Clancy C. E., Binder M. D., Santana L. F. (2019). A Stochastic Model of Ion Channel Cluster Formation
in the Plasma Membrane. J. Gen. Physiol..

[43] Sungkaworn T., Jobin M.-L., Burnecki K., Weron A., Lohse M. J., Calebiro D. (2017). Single-Molecule Imaging Reveals Receptor–G Protein
Interactions at Cell Surface Hot Spots. Nature.

[44] Elgsaeter A., Branton D. (1974). Intramembrane Particle Aggregation in Erythrocyte Ghosts. J. Cell Biol..

[45] Kasugai Y., Swinny J. D., Roberts J. D. B., Dalezios Y., Fukazawa Y., Sieghart W., Shigemoto R., Somogyi P. (2010). Quantitative Localisation
of Synaptic and Extrasynaptic GABAA Receptor Subunits on Hippocampal
Pyramidal Cells by Freeze-Fracture Replica Immunolabelling. Eur. J. Neurosci..

[46] Liu S.-L., Sheng R., Jung J. H., Wang L., Stec E., O’Connor M. J., Song S., Bikkavilli R. K., Winn R. A., Lee D., Baek K., Ueda K., Levitan I., Kim K.-P., Cho W. (2017). Orthogonal Lipid Sensors
Identify Transbilayer Asymmetry of Plasma Membrane Cholesterol. Nat. Chem. Biol..

[47] Zhang Y., MacKinnon R. (2025). Higher-Order
Transient Structures and the Principle
of Dynamic Connectivity in Membrane Signaling. Proc. Natl. Acad. Sci. U. S. A..

[48] Salavessa L., Lagache T., Malardé V., Grassart A., Olivo-Marin J.-C., Canette A., Trichet M., Sansonetti P. J., Sauvonnet N. (2021). Cytokine Receptor Cluster Size Impacts
Its Endocytosis
and Signaling. Proc. Natl. Acad. Sci. U. S.
A..

[49] Brown D. A., Rose J. K. (1992). Sorting of GPI-Anchored Proteins to Glycolipid-Enriched
Membrane Subdomains during Transport to the Apical Cell Surface. Cell.

[50] Simons K., Ikonen E. (1997). Functional Rafts in Cell Membranes. Nature.

[51] Walton J. H., Jenkins J. D. (1923). A Study Of The Ternary
System, Toluene-acetone-water. J. Am. Chem.
Soc.

[52] Tanford C. (1978). The Hydrophobic
Effect And The Organization Of Living Matter. Science.

[53] Castello-Serrano I., Lorent J. H., Ippolito R., Levental K. R., Levental I. (2020). Myelin-Associated
MAL and PLP Are Unusual among Multipass Transmembrane Proteins in
Preferring Ordered Membrane Domains. J. Phys.
Chem. B.

[54] Pérez-Mitta G., Sezgin Y., Wang W., MacKinnon R. (2024). Freestanding
Bilayer Microscope for Single-Molecule Imaging of Membrane Proteins. Sci. Adv..

[55] Schindelin J., Arganda-Carreras I., Frise E., Kaynig V., Longair M., Pietzsch T., Preibisch S., Rueden C., Saalfeld S., Schmid B., Tinevez J.-Y., White D. J., Hartenstein V., Eliceiri K., Tomancak P., Cardona A. (2012). Fiji: An Open-Source
Platform for Biological-Image Analysis. Nat.
Methods.

[56] Mandala V. S., MacKinnon R. (2025). Electric Field-Induced Pore Constriction in the Human
Kv2.1 Channel. Proc. Natl. Acad. Sci. U. S.
A..

[57] Bligh E. G., Dyer W. J. (1959). A Rapid Method of Total Lipid Extraction and Purification. Can. J. Biochem. Physiol..

[58] Vaisey G., Banerjee P., North A. J., Haselwandter C. A., MacKinnon R. (2022). Piezo1 as a Force-through-Membrane
Sensor in Red Blood
Cells. Elife.

[59] Dumelie J. G., Chen Q., Miller D., Attarwala N., Gross S. S., Jaffrey S. R. (2024). Biomolecular Condensates
Create Phospholipid-Enriched
Microenvironments. Nat. Chem. Biol..

[60] Hughes C. S., Moggridge S., Müller T., Sorensen P. H., Morin G. B., Krijgsveld J. S.-P. (2019). Solid-Phase-Enhanced
Sample Preparation for Proteomics
Experiments. Nat. Protoc..

[61] Demichev V., Messner C. B., Vernardis S. I., Lilley K. S., Ralser M. D.-N. (2020). Neural
Networks and Interference Correction Enable Deep Proteome Coverage
in High Throughput. Nat. Methods.

[62] Shannon P., Markiel A., Ozier O., Baliga N. S., Wang J. T., Ramage D., Amin N., Schwikowski B., Ideker T. (2003). Cytoscape: A Software Environment
for Integrated Models
of Biomolecular Interaction Networks. Genome
Res..

[63] Zhao C., MacKinnon R. (2023). Structural and Functional Analyses of a GPCR-Inhibited
Ion Channel TRPM3. Neuron.

[64] Wang W., Touhara K. K., Weir K., Bean B. P., MacKinnon R. (2016). Cooperative
Regulation by G Proteins and Na+ of Neuronal GIRK2 K+ Channels. Elife.

[65] del Marmol, J. I. Molecular Basis of Mechanosensitivity. Doctor of Philosophy; The Rockefeller University, 2016.

[66] Mandala V. S., MacKinnon R. (2022). Voltage-Sensor Movements in the Eag
Kv Channel under
an Applied Electric Field. Proc. Natl. Acad.
Sci. U. S. A..

[67] Angelova M. I., Dimitrov D. S. (1986). Liposome Electroformation. Faraday
Discuss. Chem. Soc..

